# Multifunctional Baicalin-Modified Contact Lens for Preventing Infection, Regulating the Ocular Surface Microenvironment and Promoting Corneal Repair

**DOI:** 10.3389/fbioe.2022.855022

**Published:** 2022-03-02

**Authors:** Yue Luo, Luying Liu, Yuzhen Liao, Ping Yang, Xiaoqi Liu, Lei Lu, Jiang Chen, Chao Qu

**Affiliations:** ^1^ Sichuan Provincial Key Laboratory for Human Disease Gene Study, The Department of Medical Genetics, The Institute of Laboratory Medicine, Sichuan Academy of Medical Sciences and Sichuan Provincial People’s Hospital, University of Electronic Science and Technology, Chengdu, China; ^2^ The Department of Ophthalmology, Sichuan Provincial People’s Hospital, University of Electronic Science and Technology of China, Chengdu, China; ^3^ Institute of Biomaterials and Surface Engineering Key Lab. for Advanced Technologies of Materials, Ministry of Education, Southwest Jiaotong University, Chengdu, China; ^4^ School and Hospital of Stomatology, Wenzhou Medical University, Wenzhou, China; ^5^ The Department of Neurosurgery, Sichuan Academy of Medical Science and Sichuan Provincial People’s Hospital, Chengdu, China

**Keywords:** baicalin, contact lens, surface modification, ocular surface microenvironment, repair injury

## Abstract

Corneal injury inevitably leads to disruption of the ocular surface microenvironment, which is closely associated with delayed epithelial cell repair and the development of infection. Recently, drug-loaded therapeutic contact lenses have emerged as a new approach to treating corneal injury due to their advantages of relieving pain, promoting corneal repair, and preventing infection. However, few therapeutic contact lenses could modulate the ocular surface’s inflammation and oxidative stress microenvironment. To address this, in this study, we covalently immobilized multifunctional baicalin (BCL), a flavon molecular with anti-inflammatory, anti-oxidative stress, and antibacterial capabilities, onto the surface of the contact lens. The BCL-modified contact lens showed excellent optical properties, powerful antibacterial properties, and non-toxicity to endothelial cells. Furthermore, the BCL-modified contact lens could significantly modulate the ocular surface microenvironment, including inhibition of macrophage aggregation and resistance to epithelium damage caused by oxidative stress. In animal models, BCL-modified corneal contact lens effectively promoted corneal epithelial cells repair. These excellent properties suggested that multifunctional BCL molecules had great application potential in the surface engineering of ophthalmic medical materials.

## Introduction

Ocular surface diseases caused by corneal injury afflict more than 23 million people worldwide (Stern et al., 2018), and are among the most common eye diseases worldwide. Generally, corneal tissue can self-healing through epithelial cell migration and proliferation when the injury is not severe (Luo et al., 2021). However, when the ocular surface has intense inflammation or oxidative stress caused by extensive epithelial damage, dry eye disease, diabetic keratopathy, etc., the self-healing of the corneal epithelial becomes difficult (Ziaei et al., 2018). This difficult-to-heal epithelial injury makes the cornea susceptible to microbial infection, which can lead to corneal ulceration, corneal perforation, and even corneal blindness (Ljubimov and Saghizadeh, 2015). Therefore, preventing microbial infection and improving the ocular surface inflammatory and oxidative stress microenvironment are critical in treating corneal injuries.

Therapeutic contact lenses have become popular in treating corneal injury in clinical practice (Zidan et al., 2018) for their functions of moisturizing, relieving pain, promoting corneal epithelial repair, and reducing corneal stroma exposure (Nosrati et al., 2020). The non-drug-loaded contact lenses, namely corneal bandage lenses, were mainly used in clinical practice to treat bullous keratitis, exposure keratitis, neurotrophic keratitis, dry eye disease with epithelial lesions, trichiasis, corneal refractive surgery, and other non-infectious corneal diseases (Zidan et al., 2018). The main disadvantage of the bandage lenses was susceptibility to infection. In recent years, a new generation of drug-loaded contact lenses with infection resistance had emerged (Huang et al., 2016; Wei et al., 2019; Liu et al., 2020; Yin et al., 2021). These contact lenses could sustainably release drugs on the ocular surface, which solves the shortcomings of low drug bioavailability and poor patient compliance in traditional eye drop treatments (Jumelle et al., 2020; Lim and Lim, 2020). However, contact lenses with a microenvironment regulating ocular surface inflammation and oxidative stress are still rarely reported.

Baicalin (BCL) is a natural extract of plant origin. Due to its unique flavonoid polyphenol structure, BCL has a variety of pharmacological activities such as antibacterial, anti-inflammatory, and antioxidant properties (Zhao et al., 2016). Therefore, BCL has been extensively studied in cardiovascular (Yu et al., 2019; Xu et al., 2020), neurology (Guo et al., 2019; Li et al., 2020), and orthopedic fields (Zhao et al., 2016; Pan et al., 2019). In ophthalmology, BCL had therapeutic effects on autoimmune uveitis (Zhu et al., 2018), age-related macular degeneration (Sun et al., 2020), diabetic retinopathy (Pan et al., 2021), etc. However, at present, there is almost a gap in the application of BCL in ophthalmic materials. Therefore, we envision that modification of BCL on the surface of contact lenses could promote corneal damage repair by preventing infection and regulating the ocular surface’s inflammatory and oxidative stress microenvironment. In recent years, mussel-inspired polydopamine (PDA) coating has become a star coating for medical materials’ surface engineering because they can easily cover almost any material. Moreover, PDA coating has good biosafety and provides many secondary reactive active sites. Recently, PDA coatings have been used to modify contact lenses and exhibited good ocular surface biocompatibility (Liu et al., 2018).

In this study, we first prepared an amino-rich polydopamine-hexane diamine (PDA-HD) coating on the surface of the contact lens. Then, BCL was covalently immobilized on the PDA-HD layer by the amidation reaction. Then, we systematically studied the BCL-modified contact lens’s optical properties and surface chemistry. After that, we systematically evaluated the epithelial cell safety, anti-oxidative stress capacity, anti-inflammatory properties, and anti-microbial activity of the BCL-modified contact lens. Finally, the ability of this contact lens to promote corneal injury repair was evaluated using a rabbit animal model. Our results indicated that the BCL-modified contact lens had the potential to prevent infection, regulate the ocular surface microenvironment, and promote corneal injury repair.

## Materials and Methods

### Materials

Neifilcon A contact lenses (Alcon^®^) were purchased from CIBA Vision Corporation (Georgia, United States). Baicalin (BCL) and Hexamethylene diamine (HD) were obtained from Shanghai Aladdin Co. Ltd. (Shanghai, China). Dopamine hydrochloride (DA) and Rhodamine B were bought from Sigma-Aldrich Co. Ltd., (Shanghai, China). Dimethylformamide (DMF) was purchased from Chengdu Kelong Chemical Co., Ltd. (Chengdu, China) N-Hydroxysuccinamide (NHS) was supplied by Rhawn Co. Ltd. (China). 1-(3-Dimethylaminopropyl)-3-ethylcarbodiimide hydrochloride (EDC) was obtained from TCI Shanghai Co. Ltd. (Shanghai, China). MicroBCA kit was purchased from Pierce Biotechnology Inc. (Rockford, United States). H_2_O_2_ was supplied by Nanjing KeyGEN BioTECH Co., Ltd. (Nanjing, China).

MH broth was bought from HopeBio Co. Ltd. (Qingdao, China). Blood agar plates and DensiCHEK Plus were obtained from bioMerieux Co. Ltd. (France). Paraformaldehyde was purchased from Wuhan Servicebio Co., Ltd. (Wuhan, China).

*Staphylococcus aureus* *(S. aureus)* and *Pseudomonas aeruginosa* (*P. aeruginosa*) were obtained from Sichuan Provincial People’s Hospital (Chengdu, China). Human corneal epithelial cells (HCECs) were supplied by BNCC Co. Ltd. (China). Mice macrophages were obtained from Guge Co. Ltd. (Wuhan, China). Japanese white rabbits (3 kg) were sourced from Chengdu Dashuo Laboratory Animal Co., Ltd. (Chengdu, China).

### Preparation of the BCL-Modified Contact Lens

**FIGURE 1:**
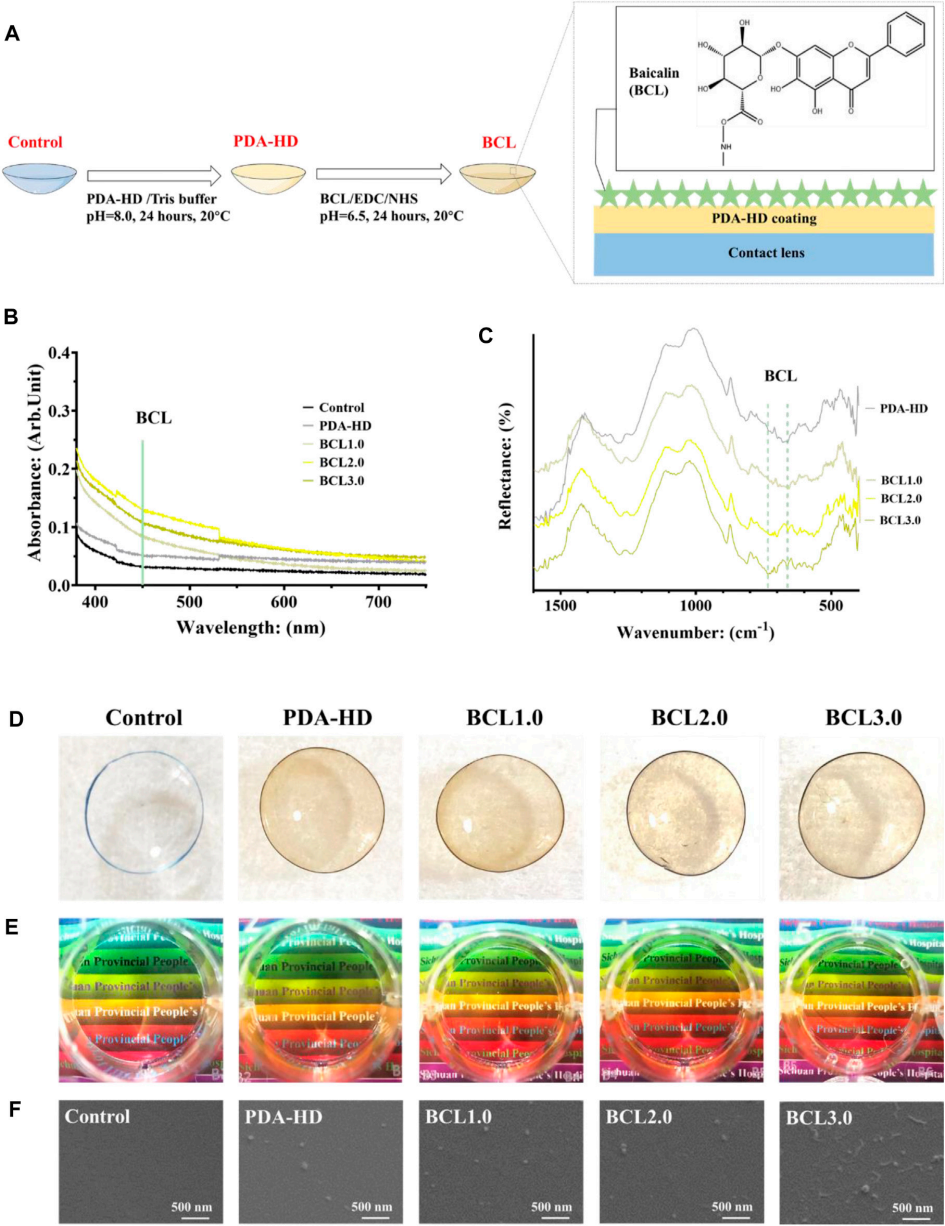
**(A)** The preparation process of BCL-modified contact lens. **(B)** UV-vis measurements of contact lenses for Control, PDA-HD, BCL1.0, BCL2.0, and BCL3.0. **(C)** FTIR measurements of contact lenses. **(D,E)** Photographs of contact lenses. **(F)** SEM images of contact lens surfaces. The untreated contact lens was used as Control.

The preparation process of the contact lens is shown in Figure 1A. The PDA-HD coating was deposited by immersing the corneal contact lens in dopamine-hexanediamine solution (DA: 1 mg/ml, Tris buffer; HD: 2.5 mg/ml; pH = 8.0, 20°C) for 24 h. After sufficient washing using distilled water, the monolayer modified PDA-HD coating of the corneal contact lens was PDA-HD.

Meanwhile, distilled water was mixed with dimethylformamide in a 1:1 volume for preparing three concentrations of BCL solutions. The concentrations of BCL were 1, 2, and 3 mg/ml, respectively. A mixture of 15.52 mg/ml EDC and 5.14 mg/ml NHS was prepared, and the BCL solutions of each concentration were activated for 15 min in a ratio of 1:10. The prepared PDA-HD-coated corneal contact lenses were immersed in the BCL solution at 20°C for 24 h. After sufficient washing with distilled water, corneal contact lenses modified with different concentrations of BCL were obtained as BCL1.0, BCL2.0, and BCL3.0, respectively.

### Characterization of the BCL-Modified Contact Lens

The surface morphology of the contact lens was characterized by scanning electron microscopy (SEM; Quanta 200, FEI, Netherlands). The optical properties of the contact lens were analyzed by a UV-visible spectrophotometer (UV-Vis; TU-1901, Persee, China). The surface structure of the contact lenses was analyzed by a Fourier transform infrared spectroscopy (NICOLET5700FT-IP, Thermoelectric co., ltd., United States). The contact lens’s surface amino and phenolic hydroxyl contents were quantified by the acid orange method and MicroBCA kit, respectively. The hydrophilicity of the contact lens was detected by a drop shape analysis system (DSA 100, Krüss, Germany) in a static manner. X-ray photoelectron spectroscopy (XPS) was performed using the XSAM800 device (Kratos Ltd., United Kingdom) to detect the surface elements of the contact lens.

### Inflammatory Properties Test *in vitro*

Peritoneal macrophages from Sprague Dawley rats were cultured on samples at a cell concentration of 6 × 10^4^/mL to test the inflammatory properties of contact lenses. After incubation at 37°C for 24 h, the culture medium was discarded, and the contact lenses were washed three times with saline and fixed for 24 h by adding 4% paraformaldehyde. Rhodamine stain was added to the surface of the contact lens, stained for 15 min for observation with an inverted fluorescence microscope, and cell adhesion density was calculated.

### Corneal Epithelial Cells Growth and H_2_O_2_ Damage

Human corneal epithelial cells (HCECs) were used for *in vitro* testing to verify their biocompatibility and resistance to oxidative stress.

The contact lenses without surface modification and the prepared contact lenses were cleaned and sterilized and then placed into a 24-well cell culture plate, cultured with HCECs for 12 h. After that, 100 μM H_2_O_2_ was added to each well for 12 h, and the medium was discarded. Then the contact lenses were thoroughly washed three times with physiological saline. The cells were stained with acridine orange/propidium iodide (AO-PI), incubated at 37°C for 3 min, and then observed with an inverted fluorescence microscope, and the cell survival rate was calculated.

### Evaluation of the Antibacterial Ability

*S. aureus* and *P. aeruginosa* were collected from the corneas of patients. Each species of bacteria was inoculated onto blood agar plates and incubated in a 37°C incubator. After the appearance of many colonies, they were eluted with saline and quantified with DensiCHEK Plus at 1 × 10^8^ CFU/mL. And 10 μL of bacterial broth was added into 0.99 ml of MH broth to each well of a 24-well plate. The final concentration of *S. aureus* and *P. aeruginosa* in each well was 3 × 10^6^ CFU/mL. The contact lenses were added to the above bacterial broth and incubated at 37°C for 24 h. Then the bacterial broth was discarded, and the contact lenses were washed three times with physiological saline and fixed with 4% paraformaldehyde for 24 h. A rhodamine stain was added to the contact lenses, stained for 15 min, and observed with an inverted fluorescence microscope. Bacterial adhesion on the surface of the corneal contact lens was observed by scanning electron microscopy (SEM) and fluorescence microscope. The ratio of bacterial adhesion area was calculated by ImageJ (National Institutes of Health, United States). The number of parallel samples tested was 3.

### *In vivo* Test of Wearing BCL-Modified Contact Lens

All animal experiments were approved by the Medical Ethics Committee of the Affiliated Hospital of University of Electronic Science and Technology & Sichuan Provincial People’s Hospital. Japanese large-eared white rabbits (3 Kg) were obtained from Chengdu Dashuo Laboratory Animal Co. All animals were acclimated for 1 week. The rabbit corneal injury model was first established. Preoperatively, the whole layer of the epithelium was damaged with a cotton swab dipped in medical alcohol. After staying for a few seconds, the damaged corneal epithelium was scraped off with the back of a scraper blade, and the scraped epithelium continued to be cleaned with a moistened cotton swab. The selected BCL-modified (BCL2.0) contact lens was worn randomly to the left or right eye of the rabbit, and then the regular contact lens was worn on the opposite side as a control. Corneal epithelial healing was observed after 1 day of wearing the contact lens. After 1 day of wearing contact lenses, corneal samples were collected from rabbits for H&E staining and corneal morphology analysis.

### Data Statistics

All the above experiments were performed in triplicate (*n* = 3), and one-sided ANOVA was performed on all experimental results using GraphPad 8.0.2, with *p* < 0.05 being a statistically significant difference.

## Results and Discussions

### Characterization of BCL-Modified Contact Lens

Figure 1A showed the preparation process of multifunctional BCL-modified contact lenses. As shown in Figure 1B, the UV-vis results showed a gentle shoulder peak at 450 nm for the BCL-modified samples emerged, implying the successful modification of BCL. In addition, the BCL-modified contact lens had a stronger absorption in the UV band than the Control, which implied that the BCL-modified contact lens had a stronger UV resistance, which had positive implications for the rehabilitation of eye diseases. Figure 1C showed FTIR measurements of contact lenses, and two BCL-specific absorption peaks could be observed around wavenumbers 660 and 740 cm^−1^.

As shown in Figure 1D, the colors of PDA-HD, BCL1.0, BCL2.0, and BCL3.0 turned slightly yellow compared to the Control. As shown in Figure 1E, when viewing the colored words through the contact lenses with surface deposited PDA-HD and different contents of BCL, on the one hand, the words were clear, which indicated good transparency of the contact lenses and could meet the daily visual needs. At the same time, the color of the colored backing of the words was easily distinguishable, which indicated that the wear of the contact lenses did not interfere with daily use, such as the discrimination of traffic lights. The above study illustrated that the multifunctional BCL-modified contact lens had an excellent optical performance.

Figure 1F showed SEM photos of several sets of contact lenses. The surfaces of PDA-HD, BCL1.0, and BCL2.0 were relatively smooth, and there were distributed nanoparticles, which might be the circular particles of PDA-HD formed during deposition (Lee et al., 2007). In the BCL3.0 sample, strip-shaped particles longer than 300 nm appeared on the surface, which might be caused by the high concentration of BCL solution during the sample preparation and the deposition of BCL agglomerates on the sample surface.

**FIGURE 2:**
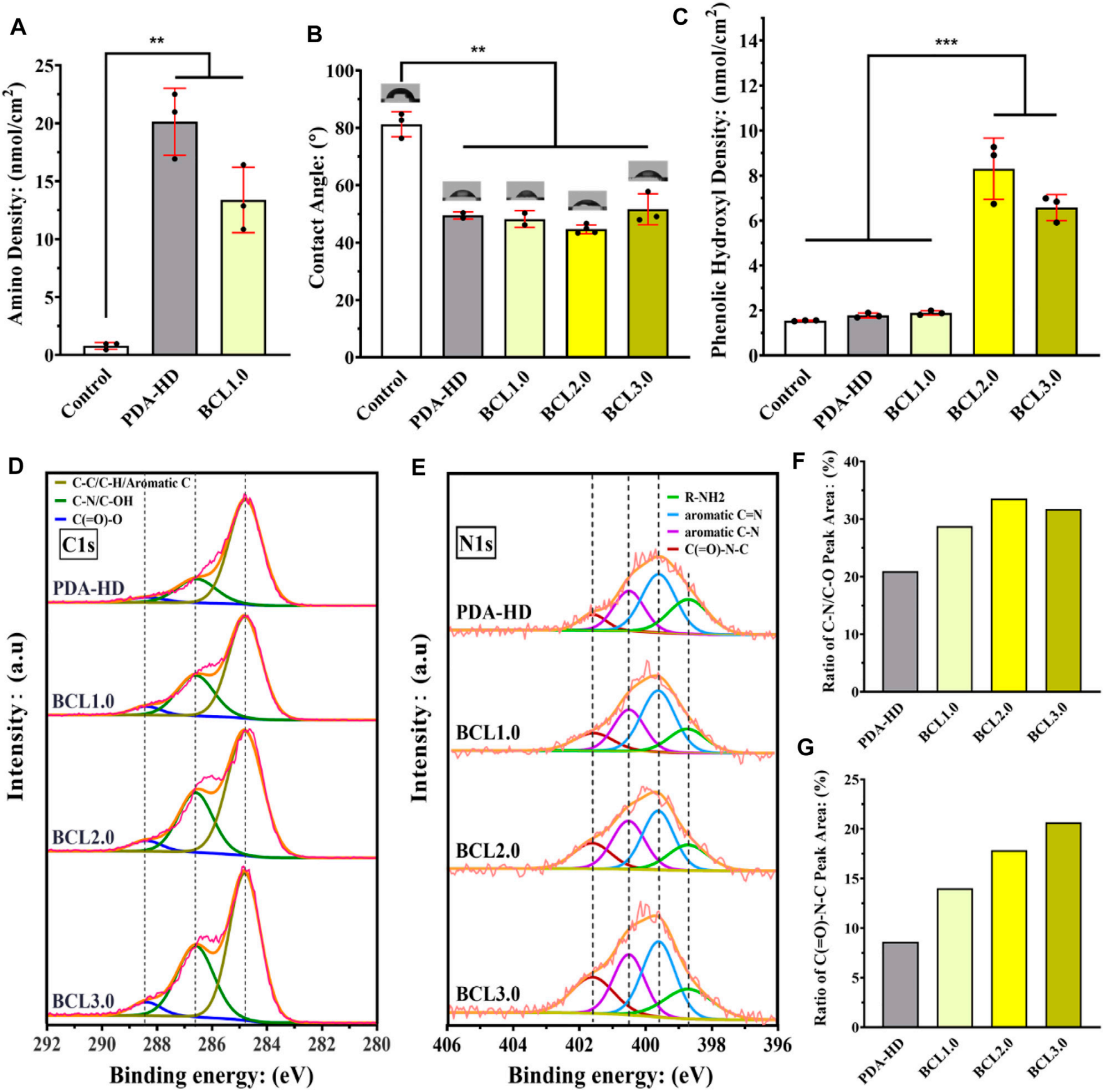
**(A)** Amino density of contact lenses surface. **(B)** Water contact angle measurement on the contact lenses surface. **(C)** Phenolic hydroxyl density on the contact lenses surface. The close view of C1s peak **(D)** and N1s peak **(E)** on the surface of the contact lenses were detected using XPS, and the C-N/C-OH peak area in the C1s peak **(F)** and the C (=O)-N-C peak area in the N1s peak **(G)** were counted. The untreated contact lenses were used as Control. Data are presented as the mean ± SD of *n* = 3 and were analyzed using one–way ANOVA, ***p < 0.001.

As shown in the results of the amino group quantification experiment in Figure 2A, there was almost no active amino group on the surface of the Control (the amino group density was about 0.79 nmol/cm^2^). In contrast, after deposition of PDA-HD, the amino density on the sample surface increased to 20.13 nmol/cm^2^, indicating a large number of amino groups on the contact lens surface after deposition of PDA-HD. Further, after grafting BCL on PDA-HD, the amino group on the BCL1.0 decreased to 13.38 nmol/cm^2^, which implies that portion of the amino group of the PDA-HD coating was chemically bound to the carboxyl group of BCL, allowing covalently immobilized BCL. Figure 2B showed the results of the water contact angle measurements of contact lenses. The water contact angle of the Control was about 81.3°, which indicated that the silicone hydrogel corneal contact lens was hydrophobic and might have poor comfort during actual wear. However, in the PDA-HD, BCL1.0, BCL2.0, and BCL3.0, the water contact angle decreased to 49.5°, 48.3°, 44.7°, and 51.6°, respectively, indicating that the PDA-HD coating improved the hydrophilicity of the contact lens (Ho and Ding, 2014), which might be beneficial to the comfort of contact lens wear.

Figure 2C showed the results of phenolic hydroxyl quantification experiments. The phenolic hydroxyl density of the Control, PDA-HD, BCL1.0, BCL2.0, and BCL3.0 was 1.55, 1.78, 1.89, 8.3, and 5.03 nmol/cm^2^, respectively. BCL2.0 had the most phenolic hydroxyl content among all BCL-modified samples, while the number of phenolic hydroxyl groups on BCL3.0 was rather inferior to that on BCL2.0. This might be due to the poor dissolution of the BCL solution used to prepare BCL3.0.

We analyzed the chemical state of the contact lenses surface using XPS. As shown in Figure 2D, the results of C1s peak detection showed that the peak at 285.6 eV (belonging to C-N or C-OH groups) increased when grafted with BCL, which implied an increase in hydroxyl content. Figure 2F showed that the area ratio of the peak of PDA-HD, BCL1.0, BCL2.0, and BCL3.0 at 285.6 eV was 20.95, 28.76, 33.59, and 31.73%, respectively. BCL2.0 had the highest elemental area ratio among all samples, which implied the most BCL molecules were modified on BCL2.0, which is consistent with the results of phenolic hydroxyl quantification experiments.

As shown in Figure 2E, the results of N1s high-resolution spectrum showed that the peak appearing at 401.5 eV represents C (=O)-N-C. The signal of this peak was significantly enhanced when the BCL was modified, suggesting the formation of the amide bond (Hao et al., 2019). Figure 2G showed the area ratio of the peak of PDA-HD, BCL1.0, BCL2.0, and BCL3.0 at 401.5 eV was 8.64, 14.02, 17.86, and 20.68%, respectively. The above results implied that BCL was covalently immobilized on the PDA-HD coating through the amide bond formation. Such covalently immobilized BCL molecules generally have excellent stability and are not easily shed by tear washout.

### The Ability of BCL-Modified Contact Lens to Modulate the Inflammatory Response

A series of immune responses often accompany corneal tissue injury or abrasion, including infiltration of inflammatory monocytes into the area of injury and differentiation into macrophage populations, which regulate tissue damage and repair, and release of pro-inflammatory cytokines and chemokines (Wynn and Vannella, 2016). The inflammatory response helps to destroy pathogens at the site of injury, but it also slows down the process of tissue repair. Therefore, controlling the unnecessary inflammatory response is beneficial to promote corneal repair. BCL had been reported to alter macrophage phenotype and attenuate the inflammatory response by inhibiting TLR4/NF-κB signaling pathway (Fu et al., 2021) and JAK/STAT pathway (Xu et al., 2020).

**FIGURE 3:**
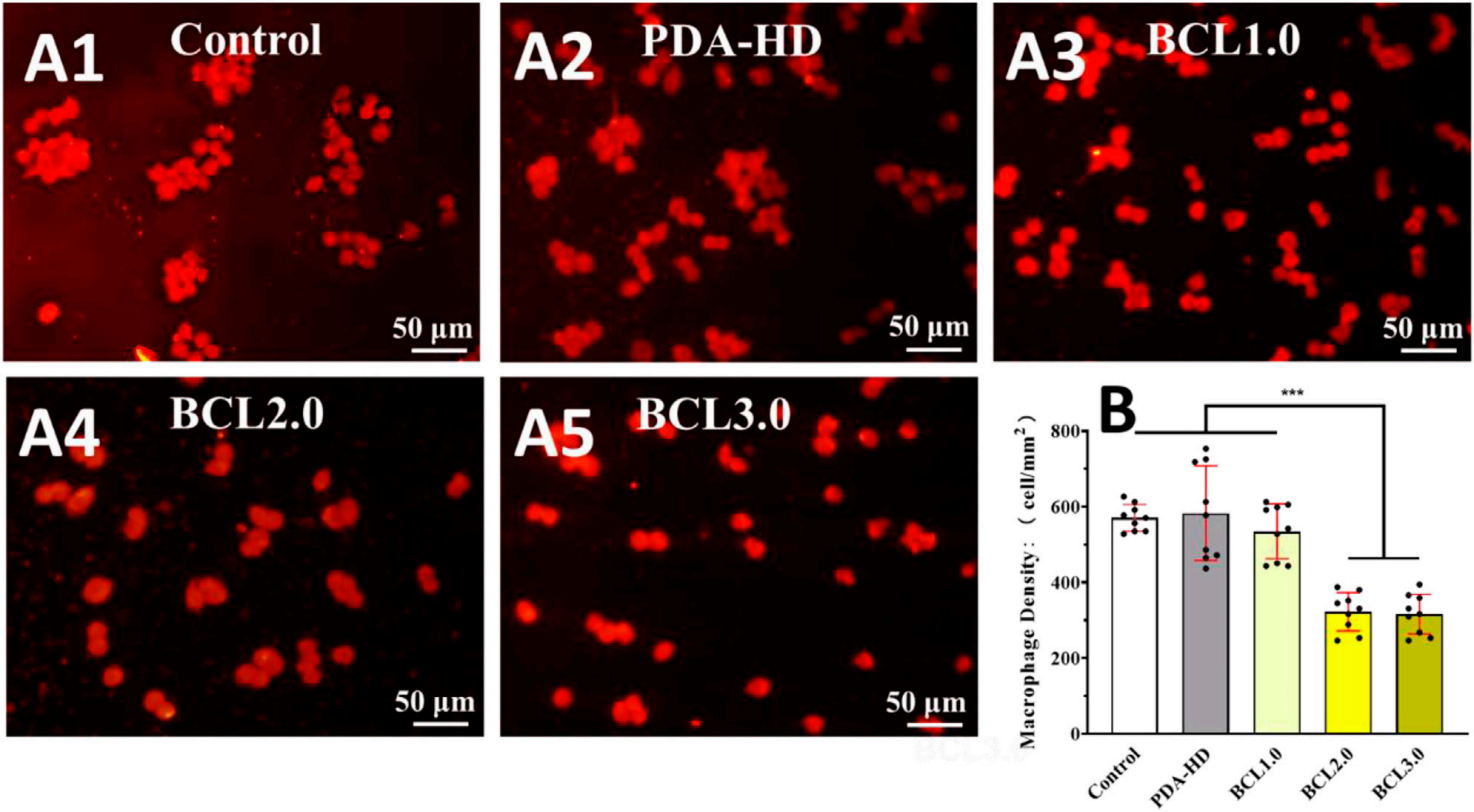
Fluorescent staining of the contact lens surface for macrophage adhesion was photographed **(A1–A5)** and counted **(B)**. The untreated contact lenses were used as Control. Data are presented as the mean ± SD of *n* = 3 and were analyzed using one–way ANOVA, ***p < 0.001.

In this study, the modulatory effect of the BCL-modified contact lenses on the inflammatory response was assessed by macrophage culture. Figures 3A1–A5 showed the growth of macrophages on the contact lenses after 24 h of culture in the high-sugar medium. Figure 3B showed the macrophage density on the contact lenses. As shown in Figure 3, the Control and PDA-HD groups had a large and aggregated number of macrophages adhering to the contact lenses. There was a slight decrease in the number of macrophages adhering to BCL1.0, but this decrease was not statistically significant. In contrast, there was a significant decrease in the number of adherent macrophages on BCL2.0 and BCL3.0. Such results suggested that the BCL modified on the contact lenses surface inhibited macrophage adhesion and exhibited anti-inflammatory properties. The above results implied that the multifunctional BCL-modified contact lenses could effectively regulate the ocular surface inflammatory microenvironment.

### The Ability of BCL-Modified Contact Lens to Modulate Oxidative Stress

Normal corneal epithelial tissue is rich in antioxidant enzymes to scavenge excess free radicals and reactive oxygen species (ROS). When the corneal epithelium is damaged, the oxidative stress response is triggered along with the activation of the inflammatory response, releasing large amounts of ROS, creating a vicious cycle of oxidative stress and inflammation (Kaluzhny et al., 2020). Therefore, there is a need to regulate the oxidative stress microenvironment of the ocular surface.

**FIGURE 4:**
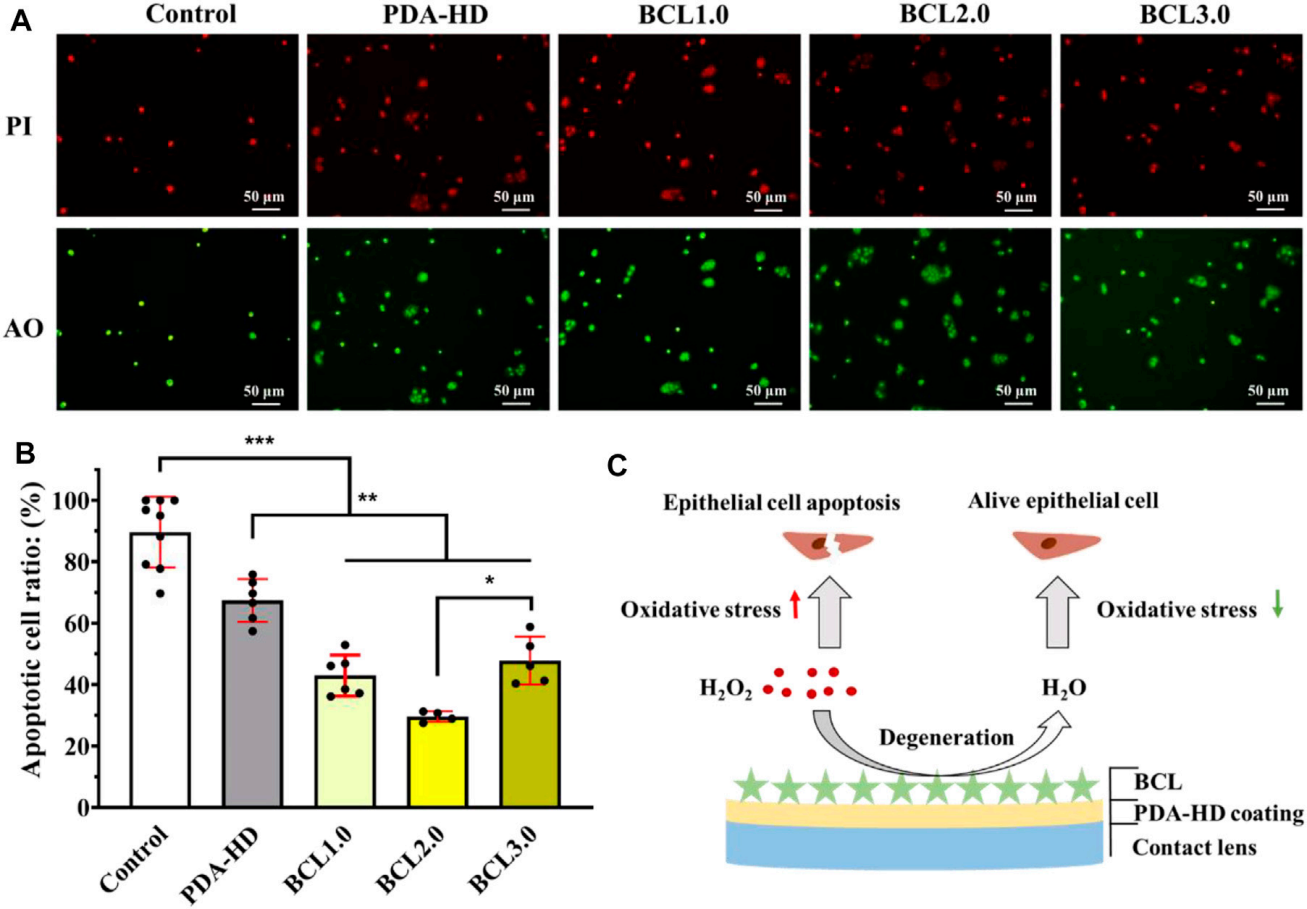
**(A)** Fluorescence staining of HCECs growth and apoptosis on corneal contact lens after addition of hydrogen peroxide was photographed. **(B)** Survival statistics of HCECs on the contact lenses. **(C)** Schematic diagram of BCL-modified multifunctional corneal contact lens catabolizing hydrogen peroxide during cell culture. The untreated contact lenses were used as Control. Data are presented as the mean ± SD of *n* = 3 and were analyzed using one–way ANOVA, *p < 0.05, **p < 0.01, ***p < 0.001.

It has been reported that BCL had a powerful antioxidant capacity due to its special polyphenolic flavonoid structure, which significantly reduced mitochondrial and intracellular ROS levels, thus acting as a protective agent for cells (Song et al., 2020). As shown in Figures 4A,B, the cell growth and apoptosis of HCECs cultured on contact lenses and added with hydrogen peroxide were identified by acridine orange (green)/propidium iodide (red) (AO/PI) staining. The results showed that on Control samples with many PI-positive cells, the apoptosis rate was 88.97%, indicating a significant killing effect of H_2_O_2_ on HCECs. While on the surface of PDA-HD, BCL1.0, BCL2.0, and BCL3.0 samples, PI-positive cells were reduced with apoptosis rates of 67.42, 42.99, 29.63, and 47.84%, respectively. This trend of apoptosis rate was consistent with the trend of phenolic hydroxyl groups on the samples. (seen in Figure 2C). The above results indicated that the multifunctional BCL-modified contact lens could effectively inhibit the damage of HCECs by ROS.

Such results were also in line with our expectations. Firstly, the dopamine molecule, a constituent of the PDA-HD coating, has a catechol structure and has been shown to have free radical scavenging ability (Hu et al., 2020). Further, we modified the flavonoid polyphenol BCL on the surface of PDA-HD, thus enhancing the ability of the coating to regulate the oxidative stress microenvironment. A schematic diagram of the BCL-modified contact lens scavenging hydrogen peroxide during cell culture was shown in Figure 4C.

### *In vitro* Antimicrobial Properties of BCL-Modified Contact Lens

After a corneal injury, the barrier is disrupted, and ocular surface homeostasis is broken, at which time the normal flora or conditionally pathogenic bacteria in the conjunctival capsule can turn into pathogenic bacteria invasion, releasing various proteases and exotoxins to enhance the inflammatory response and oxidative stress response. If the infection is further aggravated, a biofilm can form and eventually lead to severe corneal disease (Zhang et al., 2020). Therefore, it is important to repair the damage while preventing microbial infections.

**FIGURE 5:**
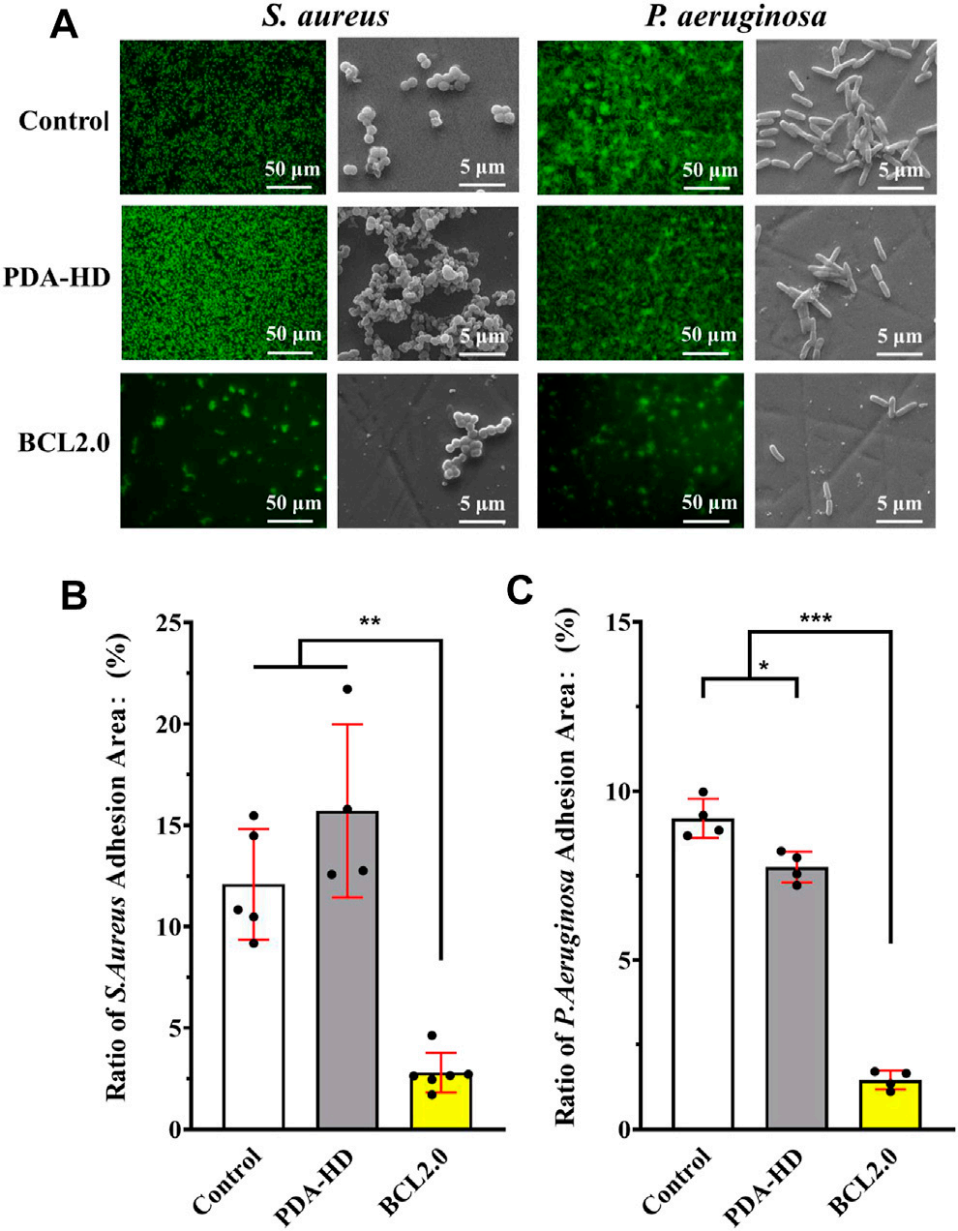
**(A)** Fluorescence stained photographic and SEM scanned images of *S. aureus* and *P. aeruginosa* cultured on the contact lenses of Control, PDA-HD, and BCL2.0 for 24 h. **(B)** Statistics of the adherence area of *S. aureus* on the surface of the contact lenses. **(C)** Statistics of the adhesion area of *P. aeruginosa* on the surface of the contact lenses. The untreated contact lenses were used as Control. Data are presented as the mean ± SD of *n* = 3 and were analyzed using one–way ANOVA, *p < 0.05, **p < 0.01, ***p < 0.001.

The results of antimicrobial experiments on contact lenses were shown in Figure 5A. After the Control, PDA-HD, and BCL2.0 contact lenses were incubated in *S. aureus* and *P. aeruginosa* bacterial solutions for 24 h, the adhesion of bacteria on the contact lenses were observed by fluorescence inverted microscopy and SEM, and the adhesion area was calculated (Figures 5B,C). The results showed that both *S. aureus* and *P. aeruginosa* adhered significantly less to the BCL2.0 surface compared to the Control. The adherence area ratios of *S. aureus* and *P. aeruginosa* were about 12.09 and 9.19% on Control samples, respectively, while they decreased to about 2.8 and 1.46% on BCL2.0, respectively., the above results indicated that the multifunctional BCL-modified contact lens had excellent antibacterial ability and was expected to prevent bacterial infections during wear.

### Effect of BCL-Modified Contact Lens on Corneal Injury Treatment

Corneal epithelial cell injury repair is a continuous and intersecting process involving epithelial cell proliferation, migration, and adhesion (Lu et al., 2001). In the early stages of trauma, necrotic shedding and progressive thinning of superficial epithelial cells around the corneal wound occurs, and flattened epithelial cells at the wound periphery extend pseudopods toward the wound surface. When the wound is close to the corneal rim, epithelial cells proliferate actively to fast repair the injury. When the wound is far from the corneal rim or located in the center of the pupil, the replenishment of epithelial cells at the wound moves centripetally from the normally proliferating epithelial cells at the corneal rim. The epithelial cells that add value and move to the wound then complete epithelial cell adhesion, i.e., tight epithelial cell-epithelial cell junctions and semi-bridging granule junctions between the epithelial cells and the basement membrane (Thill et al., 2007).

**FIGURE 6:**
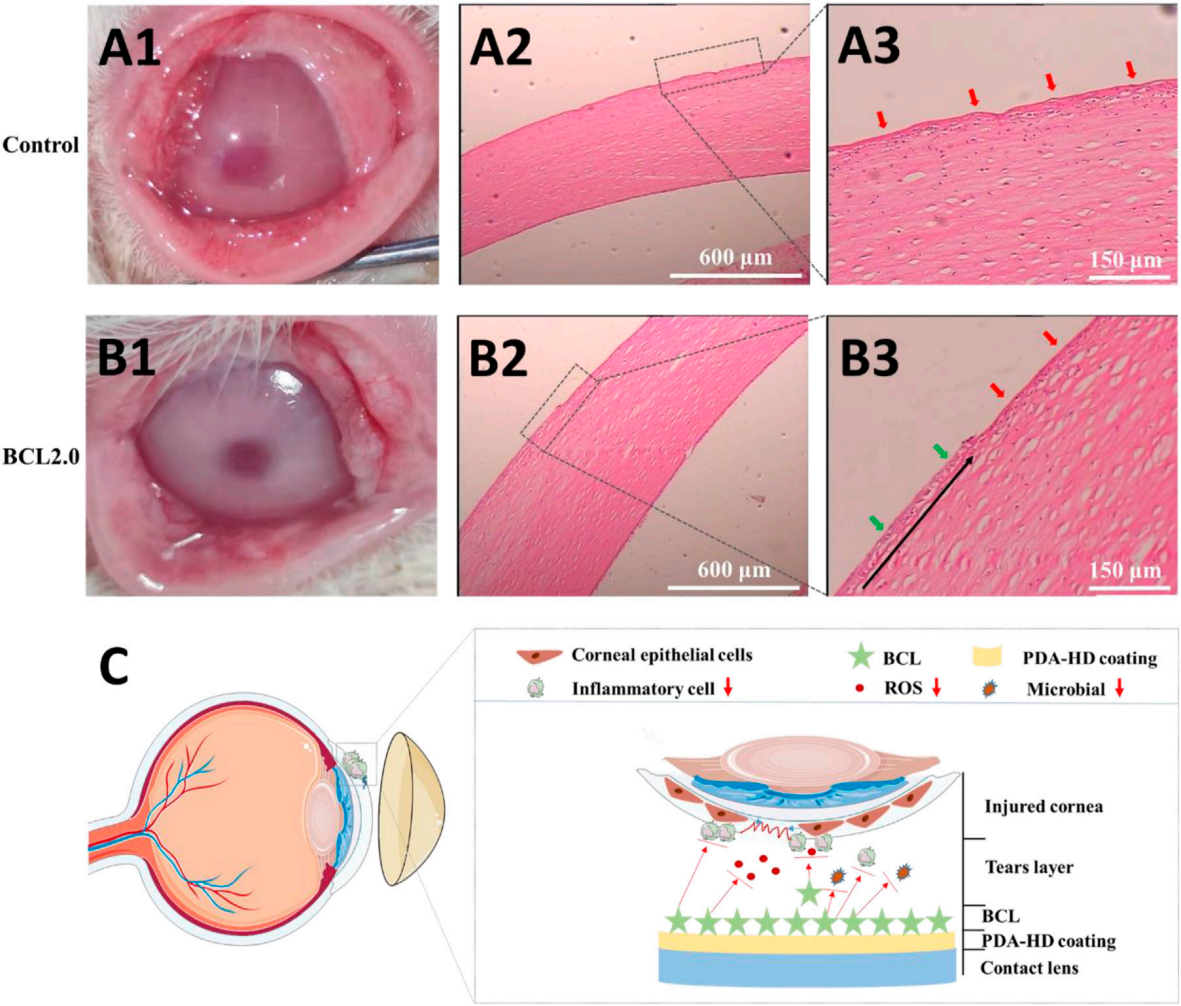
The injured rabbits’ cornea was treated by the Control or the BCL2.0 contact lenses for 24 h and observed by hematoxylin & eosin (H & E) staining. **(A1–A3)** Control group **(B1–B3)** BCL2.0 group. **(C)** Schematic diagram of the BCL-modified contact lens promoted corneal injury repair. The untreated contact lenses were used as Control.

As shown in Figure 6C, we envisioned that the BCL-modified contact lens could promote corneal damage repair by preventing infection and regulating the ocular surface’s inflammatory and oxidative stress microenvironment. We used animal experiments to verify this hypothesis.

The rabbit corneal epithelium was first disrupted and scraped in its entirety using 75% alcohol. Then the rabbit corneal morphology was observed by wearing the Control and BCL2.0 lenses for 24 h, respectively. As shown in Figures 6A1,B1, the Control lenses treated corneas were slightly cloudy, while the BCL2.0 lenses treated corneas were clean. Furthermore, more lid conjunctiva and ocular secretions were observed in the Control group than in the BCL2.0 group. These results indicated that the BCL2.0 contact lens could more effectively relieve the inflammatory response and oxidative stress in the injury corneas than the Control contact lens.

As shown in Figures 6A2,A3, the entire cornea appeared uneven in the Control group (red arrows), with many immune cells collecting between the corneal stroma. The proliferating and migrating new corneal epithelium was not observed yet. As shown in Figures 6B2,B3, compared with the Control group, fewer immune cells accumulate between the corneal stroma in the BCL2.0 group. And new corneal epithelial cells (green arrows) that were proliferating and migrating from the corneal limbus to the pupil center could be observed (the black arrow in Figure 6B3 shows the direction of corneal epithelial cell migration). The above results indicated that after a large area of corneal injury, wearing the multifunctional BCL-modified contact lenses could significantly boost the restoration of corneal injury at an early stage.

## Conclusion

In conclusion, we developed a simple and easy method to creatively covalently immobilize BCL onto a contact lens using dopamine-hexanediamine (PDA-HD) coating as a transition layer, thus endowing the contact lens with desirable multifunctions. The material science characterization showed that the BCL-modified contact lens had good optical properties and UV resistance. Besides, the BCL-modified contact lens also owned excellent hydrophilic and reductive properties. *In vitro* biological evaluation showed that the BCL-modified contact lens had epithelial cell safety and antibacterial activity. Moreover, the BCL-modified contact lens could regulate the inflammation and oxidative stress microenvironment, lacking in most current contact lenses. In the animal study, the multifunctional BCL-modified contact lens was safe to wear and promoted injury repair of corneal epithelium. Thus, the BCL-modified contact lens has potential applications in preventing infection, regulating the ocular surface microenvironment, and promoting corneal injury repair. This study provides a new option for surface engineering of therapeutic contact lenses to treat ocular surface diseases.

## Data Availability

The original contributions presented in the study are included in the article/supplementary material, further inquiries can be directed to the corresponding authors.
